# CRIP1 promotes docetaxel resistance and immune-associated cell death modulation in prostate cancer

**DOI:** 10.3389/fphar.2026.1769906

**Published:** 2026-03-31

**Authors:** Dehua Zhang, Meiling Han, Ni Yan, Yu Zhang, Chao Wang, Wenzhong Shi, Wenxue Jia, Jun Cao

**Affiliations:** 1 Department of Urology, Yiling Hospital of Yichang, Affiliated Yiling Hospital of China Three Gorges University, Yichang, Hubei, China; 2 Department of Dermatology, Yiling Hospital of Yichang, Affiliated Yiling Hospital of China Three Gorges University, Yichang, Hubei, China

**Keywords:** chemotherapy resistance, computational prioritization, Crip1, docetaxel resistance, drug target, immunogenic cell death, prostate cancer

## Abstract

**Background:**

Docetaxel resistance is a major barrier to durable disease control in advanced and castration-resistant prostate cancer. There is a pharmacological need to identify biomarkers that not only stratify resistance risk but also nominate tractable regulators whose perturbation can restore taxane sensitivity and suppress resistant phenotypes.

**Methods:**

We analyzed docetaxel-resistant prostate cancer cell models to derive resistance-associated transcriptional candidates and used computational prioritization to construct a compact, taxane-resistance–anchored gene set. Associations of the gene set and key candidates with disease progression were evaluated in TCGA-PRAD, which predominantly represents treatment-naïve primary tumors and therefore provides progression relevance rather than treatment-specific response validation. Docetaxel-resistant cell lines were established for functional validation, and CRIP1 was stably silenced to assess effects on drug sensitivity, clonogenic growth, migration, apoptosis, and immune-associated cell-death features. In addition, an LNCaP-DTXr xenograft model was used to evaluate the impact of CRIP1 knockdown on docetaxel response *in vivo*.

**Results:**

A three-gene, taxane-resistance–anchored signature was derived and showed progression-related associations in TCGA-PRAD. Among candidates, cysteine-rich protein 1 (CRIP1) was consistently upregulated in resistant models and emerged as a top resistance-associated factor. Functionally, CRIP1 knockdown restored docetaxel sensitivity, reduced clonogenic survival and migratory capacity, and enhanced docetaxel-induced apoptosis in resistant prostate cancer cells. Consistently, CRIP1 depletion significantly suppressed tumor growth and reduced tumor burden in docetaxel-treated LNCaP-DTXr xenografts, indicating restored chemosensitivity *in vivo*. In parallel, CRIP1 depletion was accompanied by changes in damage-associated and immune-related cell-death readouts under taxane stress, suggesting a potential role in linking drug tolerance to immune-relevant cell-death programs.

**Conclusion:**

These findings identify CRIP1 as a functionally validated, pharmacologically relevant mediator of docetaxel resistance in prostate cancer. While independent validation in taxane-treated clinical cohorts is warranted, our results support CRIP1 as a candidate therapeutic target and provide a mechanistic framework connecting taxane resistance with immune-associated cell-death modulation.

## 1 Introduction

According to the most recent estimates from the American Cancer Society (2025), approximately 313,780 new cases of prostate cancer are expected to be diagnosed in the United States this year, with an estimated 35,770 deaths attributed to the disease. Prostate cancer remains one of the most commonly diagnosed malignancies among men and is the second leading cause of cancer-related death behind lung cancer in this population (Sung et al., 2021; Yan Z. et al., 2025). Androgen-deprivation therapy (ADT) is initially effective for most patients, but a substantial proportion eventually progress to castration-resistant prostate cancer (CRPC), a stage characterized by biochemical and radiographic progression despite castrate testosterone levels and associated with poor long-term outcomes (Higano, 2014). For men with metastatic CRPC (mCRPC), taxane-based chemotherapy—particularly docetaxel, and later cabazitaxel—has been established as a backbone systemic therapy that modestly extends overall survival compared with older regimens (e.g., mitoxantrone plus prednisone) (Tannock et al., 2004; de Bono et al., 2010). However, primary and acquired resistance to taxanes is common, and once resistance emerges, therapeutic options are limited and prognosis remains dismal, underscoring the need for robust biomarkers and druggable nodes that link taxane response with disease progression.

Over the past decade, transcriptomic profiling of prostate tumors and experimental models has enabled the discovery of gene signatures that stratify recurrence risk and treatment response, and machine-learning approaches are increasingly used to refine these signatures and improve prognostic performance (Li et al., 2022; Kornberg et al., 2019; Cullen et al., 2015). Nonetheless, most existing models are built directly from bulk tumor cohorts such as TCGA and focus on biochemical relapse or overall survival, without explicitly anchoring the signature to drug-resistance biology in preclinical systems. At the same time, taxane-resistant CRPC cell line models—generated by long-term docetaxel or cabazitaxel exposure—have been widely used to dissect mechanisms of resistance, including alterations in microtubule dynamics, drug efflux, apoptosis evasion, and stress-response signaling (Lombard et al., 2021; Se et al., 2020; Gjyrezi et al., 2020; Duran et al., 2015). Integrating convergent transcriptional programs derived from resistance models with progression-free interval (PFI) data from patient cohorts provides a strategy to identify biomarkers that are both mechanistically linked to chemoresistance and directly informative of clinical outcomes, thereby aligning with the objectives of biomarker- and drug-target–driven precision oncology.

Cysteine-rich intestinal protein 1 (CRIP1) is a LIM-domain zinc-binding protein that has recently attracted attention as a multifunctional regulator in cancer biology (Cousins and Lanningham-Foster, 2000; Paul et al., 2023; Zhang et al., 2019). CRIP1 is overexpressed in colorectal cancer, where it suppresses apoptosis, promotes migration and invasion, and contributes to resistance to 5-fluorouracil–based chemotherapy (Zhang et al., 2019; He et al., 2017; He et al., 2019). In breast cancer, CRIP1 has been reported in association with HER2 and adverse clinicopathologic features, suggesting a context-dependent oncogenic role (Ludyga et al., 2013; Rauser et al., 2010; Sun et al., 2021). More broadly, the CRIP family has been implicated in tumorigenesis and immune regulation across multiple malignancies, yet the specific contribution of CRIP1 to prostate cancer progression, taxane resistance, and treatment-induced cell death remains poorly understood.

In parallel, there is growing interest in how chemotherapeutic agents interact with the antitumor immune response, including the concept of ICD, in which dying cancer cells emit danger-associated molecular patterns such as HMGB1 that promote dendritic-cell activation and T-cell priming (Apetoh et al., 2007; Obeid et al., 2007). Taxanes have been suggested to modulate tumor–immune interactions in addition to their direct cytotoxic effects, raising the possibility that resistance pathways may not only blunt chemotherapy-induced apoptosis but also dampen ICD-related signals, thereby reinforcing an immune-evasive niche. Yet, ICD-linked biomarkers have rarely been integrated with resistance-anchored prognostic signatures in CRPC.

In this study, we integrated two independent docetaxel-resistant CRPC cell line datasets to define a consensus transcriptional program associated with taxane resistance and projected this program onto the TCGA-PRAD cohort to identify genes linked to progression-free interval (PFI). Through a systematic multi-algorithm machine-learning framework, we established a parsimonious three-gene PFI signature (CRIP1, GLS2, and MTUS1) that achieves a balance between prognostic performance and model simplicity and demonstrates robustness across resampling procedures. We subsequently prioritized CRIP1 as a candidate driver of chemoresistance and malignant phenotypes, and performed functional assays in docetaxel-resistant LNCaP and 22Rv1 sublines to evaluate its roles in proliferation, migration, docetaxel responsiveness, apoptosis, and HMGB1-associated immunogenic cell death markers. By integrating taxane-resistant experimental models, patient-level survival data, and mechanistic validation of a candidate target, this work aligns with the objectives of the Research Topic by proposing both a clinically relevant biomarker signature and a tractable molecular node with potential to inform strategies aimed at overcoming taxane resistance in CRPC.

## 2 Methods

### 2.1 Data acquisition, preprocessing, and transcriptomic profiling across GEO and TCGA cohorts

Public multi-omics datasets used in this study were obtained from GEO and TCGA to characterize the transcriptional heterogeneity associated with docetaxel resistance. GEO datasets GSE33455 and GSE158494, each containing parental prostate cancer cell lines and their corresponding docetaxel-resistant derivatives, were downloaded using GEOquery (Davis and Meltzer, 2007), and normalized expression matrices were extracted directly from the Series Matrix files. Clinical and sample metadata were simultaneously retrieved to ensure accurate group definition (Parent vs. Resistant). TCGA-PRAD RNA-seq data (STAR-aligned gene-level counts) and corresponding clinical annotations were downloaded from the Genomic Data Commons and curated to retain 497 primary prostate adenocarcinoma patients with complete progression-free interval (PFI) information (Colaprico et al., 2016). Raw counts were later processed as described below to obtain log_2_-transformed normalized expression profiles. Principal component analysis (PCA) and uniform manifold approximation and projection (UMAP) were performed using the prcomp function and the R package umap, respectively. PFI time and event variables were constructed following TCGA Pan-Cancer clinical definitions, and the overall PFI landscape was visualized through Kaplan–Meier estimation using survminer (Liu et al., 2018). Additional clinical phenotypes—age, histological type, tumor status, margin status, and staging—were summarized by density distribution, categorical barplots, or radar charts to illustrate the clinical composition of TCGA-PRAD. Differentially expressed genes (DEGs) for both GEO datasets were computed using the limma framework (Ritchie et al., 2015) (Res vs. Parent), and probe annotations were harmonized to gene symbols using GPL platform files. Overlapping gene sets between datasets were identified through direct gene symbol intersection, and log2 fold-change values were used to evaluate cross-dataset transcriptional consistency via Pearson correlation analysis. All visualizations were generated with ggplot2, and high-resolution PDF files were exported for figure assembly.

### 2.2 Differential expression analysis and visualization

Differential expression analysis was conducted separately in GSE33455 and GSE158494 using the limma framework by contrasting docetaxel-resistant samples against parental controls (Resistant vs. Parent). For each dataset, gene-level statistics were computed using empirical Bayes moderation, and multiple testing was corrected using the Benjamini–Hochberg procedure. Genes were defined as differentially expressed if they satisfied |log2 fold-change (log2FC)| ≥ 1 and adjusted P value (FDR) < 0.05. Volcano plots were generated to display the global distribution of effect sizes and statistical significance, with upregulated, downregulated, and non-significant genes highlighted in distinct colors. For heatmap visualization (Gu et al., 2016), the top 50 DEGs were ranked by significance (FDR) and effect size (|log2FC|), and their expression profiles were plotted after Z-score scaling across samples. Samples were annotated by group (Parent vs. Resistant) to facilitate visual comparison between conditions.

### 2.3 Functional enrichment analysis of consensus DEGs

To characterize functional programs consistently associated with docetaxel resistance, overlapping DEGs between GSE33455 and GSE158494 were obtained by intersecting gene symbols passing the significance thresholds in both datasets. Gene symbols were mapped to Entrez Gene identifiers using org. Hs.e.g., db, and enrichment analyses were performed using clusterProfiler (Yu et al., 2012). Gene Ontology (GO) enrichment was conducted for Biological Process (BP), Cellular Component (CC), and Molecular Function (MF) categories with Benjamini–Hochberg adjustment; pathways with FDR <0.05 were considered significant (Ashburner et al., 2000). Kyoto Encyclopedia of Genes and Genomes (KEGG) pathway enrichment (Ogata et al., 1999) was performed similarly, and results were visualized as bubble plots, where dot size indicates the number of mapped genes (Count), dot color represents −log10(FDR), and the x-axis indicates GeneRatio.

### 2.4 Prognostic screening and candidate definition (TCGA–PRAD)

To translate the GEO-derived consensus genes into clinically relevant prognostic markers, we performed survival analyses in the TCGA–PRAD cohort using progression-free interval (PFI) as the primary endpoint (Liu et al., 2018). Gene expression values were standardized on a per-gene basis (z-score across patients) to make hazard ratios comparable across genes. Each gene was tested by univariate Cox proportional hazards regression (Abd ElHafeez et al., 2021), and multiple testing was controlled using the Benjamini–Hochberg (BH) procedure (Benjamini et al., 2001). Genes passing the prespecified FDR threshold were retained as prognostic candidates for model construction.

### 2.5 Model selection, stability assessment, and risk-score construction

We implemented a systematic model-selection framework to balance predictive accuracy and signature parsimony. Candidate genes were prioritized using feature-ranking strategies (univariate Cox ranking and LASSO-based ranking) (Simon et al., 2011), and a grid search was performed over gene-set size (K). For each configuration, we fit a multivariable Cox model and evaluated discrimination using time-dependent AUC at 3 and 5 years and their average (MeanAUC); a parsimony-aware score was additionally used to favor compact signatures (Blanche et al., 2013; Heagerty et al., 2000; Heagerty and Zheng, 2005). Feature stability was assessed via repeated resampling, recording selection frequency across repeats to identify consistently retained genes (Sauerbrei and Schumacher, 1992). The final signature was refit in a multivariable Cox model using z-scored expression, and an individual risk score was computed as a weighted linear combination of standardized expression values using the fitted coefficients. Patients were stratified into risk groups according to the predefined cutoff, and prognostic separation was evaluated using Kaplan-Meier analysis with log-rank testing (Rich et al., 2010).

### 2.6 Expression validation across GEO docetaxel-resistance cohorts

Two independent docetaxel-resistance GEO cohorts (GSE33455 and GSE158494) were analyzed using limma to estimate differential expression between resistant and parental/controls. For each cohort, log2 fold-change (Resistant − Parent) and its uncertainty were extracted for CRIP1, GLS2, and MTUS1. Cross-cohort consistency was summarized using a simple fixed-effect meta-analysis to obtain a pooled effect estimate and 95% confidence interval.

### 2.7 TCGA expression processing and pathway association analysis

For TCGA–PRAD, STAR-count expression data were converted to CPM and log2(CPM+1) values (Robinson et al., 2010). Ensembl IDs were mapped to gene symbols and duplicated symbols were aggregated. The 3-gene risk score was computed using z-scored gene expression and coefficients from the multivariable Cox model described in the prognostic modeling section. Hallmark pathway activity (Hänzelmann et al., 2013; Liberzon et al., 2015) was quantified at the sample level and then associated with the continuous risk score using Spearman correlation; multiple testing was controlled using the Benjamini–Hochberg procedure. Co-expression among CRIP1, GLS2, and MTUS1 was evaluated by Spearman correlation across tumor samples.

### 2.8 Cell culture and resistant cell line development

The human prostate cancer cell lines LNCaP and 22Rv1 were purchased from the American Type Culture Collection (ATCC) and maintained in RPMI-1640 medium supplemented with 10% Fetal Bovine Serum (FBS), 50 U/mL penicillin/50 μg/mL streptomycin and 2 mM L-glutamine (Invitrogen). Cells were incubated in a humidified atmosphere containing 5% CO_2_ at 37 °C and were used during the logarithmic growth phase for all experiments. Docetaxel-resistant sublines of LNCaP and 22Rv1 cells were generated through a repeated pulse-selection protocol modified from a previously described method. Parental cells were seeded at 60 to 70 percent confluence and exposed to 4 nM docetaxel (DTX) for 24 h. Following drug exposure, the surviving cells were washed, transferred into fresh DTX-free medium, and allowed to recover for 3 weeks to ensure restoration of robust proliferation (O'Neill et al., 2011).

After the initial cycle, cells were subjected to a second DTX challenge at 8 nM for 24 h, followed by an identical 3-week drug-free recovery period. A final exposure step was performed using 16 nM DTX for 8 h. This three-stage selection process (4 nM → 8 nM → 16 nM) was repeated four consecutive times to progressively enrich for cell populations with stable docetaxel tolerance. During selection, cell viability and proliferation recovery were routinely evaluated to monitor the acquisition of resistance.

After completing all selection rounds, the resulting resistant lines were expanded, authenticated, and cryopreserved in batches. Cells used for subsequent experiments were maintained in regular drug-free medium for at least two passages before use to avoid acute drug effects. The generated resistant sublines are referred to as LNCaP-DTXr and 22Rv1-DTXr, whereas the unselected parental cells cultured in parallel without drug exposure served as matched controls.

### 2.9 RNA extraction, reverse transcription, and quantitative real-time PCR

Total RNA was isolated from cultured cells using the TRIzol reagent (Thermo Fisher Scientific) following the manufacturer’s protocol, and RNA integrity was confirmed by spectrophotometric analysis and agarose gel electrophoresis. For reverse transcription, 1 μg of purified RNA was converted into cDNA using the PrimeScript RT reagent kit (Takara) in a 20 μL reaction mixture under the recommended cycling parameters. The resulting cDNA was diluted and used immediately for quantitative analysis. Quantitative real-time PCR was performed on a QuantStudio 5 Real-Time PCR System (Applied Biosystems) using SYBR Green chemistry (Beyotime). Each reaction consisted of cDNA template, 2× SYBR Green Master Mix, and gene-specific primers, with a total volume of 20 μL per well. Reactions were carried out in triplicate. Thermal cycling conditions were as follows: initial denaturation at 95 °C for 3 min, followed by 40 cycles of 95 °C for 10 s and 60 °C for 30 s. Melt-curve analysis was included to verify amplification specificity. GAPDH served as the internal reference gene. Relative transcript abundance was calculated using the 2^{−ΔΔCt}^ method, where ΔCt was defined as the difference between the Ct values of the target gene and GAPDH, and ΔΔCt was obtained by normalizing each ΔCt to that of the designated control sample. Fold-change values represent expression levels relative to the control group. The primer sequences used in this study were as follows:

GAPDH-F: TTC​TTT​TGC​GTC​GCC​AGC​C.

GAPDH-R: TCC​CGT​TCT​CAG​CCT​TGA​C.

CRIP1-F: AAA​CCC​TAC​TGC​AAC​CAC​CC.

CRIP1-R: ATT​AGG​GGC​AAC​AAG​GGA​GC.

### 2.10 Cell viability assay (CCK-8)

Cell viability was assessed using the Cell Counting Kit-8 (CCK-8; Beyotime). LNCaP-DTXr and 22Rv1-DTXr cells stably expressing shNC or shCRIP1 constructs (shCRIP1-1, shCRIP1-2, shCRIP1-3) were seeded into 96-well plates at a density of 4 × 10^3^ cells per well and allowed to adhere overnight. The following day, cells were exposed to docetaxel at concentrations corresponding to their established resistance levels: 5 nM for LNCaP-DTXr cells and 2 nM for 22Rv1-DTXr cells. Untreated wells served as the baseline control. Cell viability was measured at 0, 24, 48, 72, and 96 h after drug administration. At each time point, 10 μL of CCK-8 reagent was added to each well and incubated for 1.5 h at 37 °C protected from light. Absorbance at 450 nm was recorded using a microplate spectrophotometer (BioTek Epoch). Background controls containing medium and CCK-8 without cells were subtracted from all readings. Each condition was tested in triplicate wells and repeated in at least three independent experiments. Cell viability was expressed as OD450 values, and growth curves were generated in GraphPad Prism 9 based on the raw absorbance measurements.

### 2.11 Cell colony formation

The long-term proliferative capacity of docetaxel-resistant cells was evaluated by a colony formation assay. LNCaP-DTXr and 22Rv1-DTXr cells stably expressing shNC or shCRIP1 (shCRIP1-1, shCRIP1-2, shCRIP1-3) were seeded into 6-well plates at a density of 500–800 cells per well in complete medium and allowed to attach overnight. The next day, cells were exposed to docetaxel at 5 nM for LNCaP-DTXr or 2 nM for 22Rv1-DTXr and maintained under these conditions for 10–14 days, with drug-containing medium refreshed every 3 days. At the end of the incubation period, colonies were gently washed with PBS, fixed with 4% paraformaldehyde for 15–20 min, and stained with 0.1% crystal violet solution for 20–30 min at room temperature. Plates were rinsed with water and air-dried, and colonies containing ≥50 cells were counted manually or using ImageJ software. Each condition was set up in triplicate and repeated in at least three independent experiments, and the average colony number per well was used for statistical analysis.

### 2.12 Wound healing assay

Cell migratory capacity was examined using a wound healing assay. LNCaP-DTXr and 22Rv1-DTXr cells stably expressing shNC or shCRIP1 constructs (shCRIP1-1, shCRIP1-2, shCRIP1-3) were seeded into 6-well plates and cultured until a confluent monolayer (approximately 90%–100%) was established. A straight, uniform scratch was generated across the cell monolayer using a sterile 200-μL pipette tip, followed by two gentle washes with PBS to remove detached cells. Cells were then maintained in serum-reduced medium (1%–2% FBS) to minimize proliferation-driven closure. Images of the wound area were captured at 0 h and 48 h using a phase-contrast microscope (Nikon Eclipse series). For each well, at least three predefined fields were recorded. Wound closure was quantified by measuring the wound width at each time point using ImageJ software. Cell migration was expressed as the percentage of wound closure relative to the initial wound width. Migration rate was calculated as:

$$\text{migration rate }\left(\%\right)=\left[\left(\text{initial wound width}-\text{wound width at }48\;h\right)\;/\text{ initial wound width}\right]\times 100$$

Each experimental group was analyzed in triplicate and repeated in three independent biological experiments. Quantified migration rates were used for statistical analyses and graphical representation.

### 2.13 Apoptosis analysis by flow cytometry

Apoptosis was quantified using an Annexin V–FITC/propidium iodide (PI) staining assay. LNCaP-DTXr and 22Rv1-DTXr cells stably expressing shNC or shCRIP1 shRNAs (shCRIP1-1, shCRIP1-2, shCRIP1-3) were seeded into 6-well plates at 2–3 × 10^5^ cells per well and cultured overnight. For apoptosis assays, docetaxel concentrations were selected as sub-IC_50_ doses to minimize excessive cytotoxicity and to enable detection of treatment-induced apoptotic responses under sensitizing conditions. Cells were then exposed to docetaxel (25 nM for LNCaP-DTXr and 6 nM for 22Rv1-DTXr) for 48 h under standard culture conditions. After treatment, both floating and adherent cells were collected, gently washed twice with ice-cold PBS, and resuspended in 1× binding buffer at a concentration of approximately 1 × 10^6^ cells/mL.

For each sample, 100 μL of cell suspension was incubated with 5 μL Annexin V–FITC and 5 μL PI from a commercial apoptosis detection kit (BD Biosciences) for 15 min at room temperature in the dark, followed by the addition of 400 μL binding buffer. Samples were analyzed within 1 h on a flow cytometer (BD FACSCanto II). Cell populations were classified as viable (Annexin V^−^/PI^−^), early apoptotic (Annexin V^+^/PI^−^), late apoptotic (Annexin V^+^/PI^+^), or necrotic (Annexin V^−^/PI^+^). The apoptotic rate was calculated as the sum of early and late apoptotic cells. Each condition was examined in triplicate in at least three independent experiments, and data were processed using FlowJo software (version 10).

### 2.14 Flow cytometric detection of HMGB1 as a marker of ICD

HMGB1 surface expression was quantified by flow cytometry to assess ICD. LNCaP-DTXr and 22Rv1-DTXr cells stably expressing shNC or shCRIP1 shRNAs (shCRIP1-1, shCRIP1-2, shCRIP1-3) were seeded into 6-well plates and allowed to adhere overnight. Cells were then treated with docetaxel at 25 nM for LNCaP-DTXr or 6 nM for 22Rv1-DTXr for 48 h. Following treatment, both floating and adherent cells were collected, washed twice with ice-cold PBS, and resuspended in staining buffer (PBS supplemented with 1% BSA). For surface HMGB1 detection, 1 × 10^6^ cells were incubated with the Alexa Fluor® 647–conjugated anti-HMGB1 antibody (clone EPR3507; Abcam) at the manufacturer-recommended dilution for 30 min at 4 °C in the dark. After staining, cells were washed twice with staining buffer and resuspended in 300 μL buffer for analysis. Unstained and isotype controls were included for gating and compensation. Flow cytometry was performed on a BD FACSCanto II cytometer, and fluorescence intensity in the APC/Alexa Fluor 647 channel was recorded. HMGB1-positive cells were quantified based on the fluorescence threshold established from control samples. Data were analyzed using FlowJo software (version 10), and the percentage of HMGB1-positive cells was calculated for each experimental group. All experiments were conducted in triplicate with at least three independent biological replicates.

### 2.15 Xenograft tumor formation assay

The impact of CRIP1 knockdown on docetaxel sensitivity *in vivo* was evaluated using LNCaP-DTXr xenografts. LNCaP-DTXr cells stably transduced with control shRNA (shNC) or CRIP1 shRNA (shCRIP1) were collected in the logarithmic growth phase, washed with cold PBS, and resuspended in a 1:1 mixture of serum-free medium and Matrigel at a concentration of 5 × 10^7^ cells/mL. Male BALB/c nude mice (4–6 weeks old) were randomly assigned to two cohorts and subcutaneously injected in the right flank with 5 × 10^6^ shNC cells or 5 × 10^6^ shCRIP1 cells in a total volume of 100 μL. Tumor formation was monitored twice weekly. Once the average tumor volume reached approximately 100–150 mm^3^, all mice in both cohorts received the same chemotherapy regimen, consisting of docetaxel at 10 mg/kg administered intraperitoneally once per week for the duration of the experiment. Tumor length (L) and width (W) were measured with calipers every 3–4 days, and tumor volume was calculated using the formula: V = (L × W^2^)/2.

At the experimental endpoint, or when tumors in the control group approached the humane limit (maximum tumor volume ≤1,500 mm^3^), mice were sacrificed, and xenografts were excised, photographed, and weighed. Tumor growth curves were generated from serial volume measurements, and final tumor weights were used to compare the response to docetaxel between shNC- and shCRIP1-derived xenografts. All procedures were approved by the institutional animal care and use committee and conducted in accordance with relevant animal welfare guidelines. Mice were humanely euthanized using carbon dioxide (CO_2_) inhalation, with CO_2_ introduced at a rate of approximately 40% of the chamber volume per minute until respiration ceased, followed by cervical dislocation to ensure death.

### 2.16 Statistical analysis

All statistical analyses were performed using R (version 4. x) and GraphPad Prism 9.0. For GEO and TCGA transcriptomic data, differential expression, enrichment, and survival analyses were carried out as described above. Multiple testing in high-throughput analyses was controlled using the Benjamini–Hochberg procedure, and unless otherwise specified, a false discovery rate (FDR) < 0.05 was considered statistically significant. Time-dependent receiver operating characteristic (ROC) curves and Cox proportional hazards models were used to evaluate the prognostic performance of gene signatures, and results are reported as hazard ratios (HR) with 95% confidence intervals (CI).

For cell-based experiments, data are presented as the mean ± standard deviation (SD) from at least three independent biological replicates. Comparisons between groups were performed using two-tailed unpaired Student’s t-tests or one-way analysis of variance (ANOVA) followed by Tukey’s *post hoc* test, as appropriate. When the assumptions of normality or homogeneity of variance were not met, corresponding non-parametric tests (such as the Mann–Whitney U test) were used. A two-sided P value <0.05 was considered statistically significant for *in vitro* experiments.

## 3 Results

### 3.1 Overview of transcriptomic heterogeneity and clinical characteristics across GEO and TCGA cohorts

**FIGURE 1:**
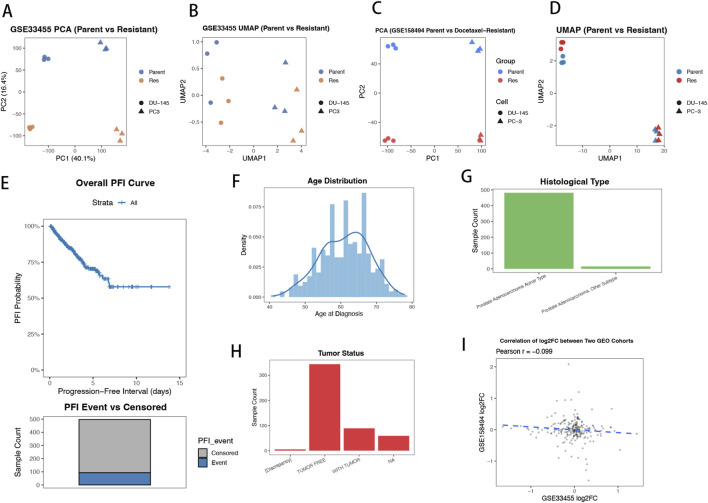
Overview of docetaxel-resistant transcriptional programs and clinical landscape in prostate cancer. **(A,B)** PCA and UMAP of GSE33455 showing clear separation between parental and docetaxel-resistant (Res) samples. **(C,D)** PCA and UMAP of GSE158494 demonstrating reproducible clustering of Parent vs. Res groups across a second GEO cohort. **(E)** Kaplan–Meier curve depicting progression-free interval (PFI) distribution in the TCGA-PRAD cohort (n = 497). **(F–H)** Clinical phenotype distributions in TCGA-PRAD, including age, histological subtype, tumor status, margin status and related variables. **(I)** Cross-dataset correlation of log2 fold-changes between GSE33455 and GSE158494, illustrating consistent resistance-associated transcriptional alterations across GEO datasets.

To characterize the global transcriptional landscape associated with docetaxel resistance, we first examined two independent GEO datasets derived from AR-null prostate cancer cell line models. In GSE33455, both PCA and UMAP analyses (Figures 1A,B) demonstrated clear separation between parental and resistant cell lines, indicating extensive transcriptomic reprogramming upon resistance acquisition. A similar pattern was independently reproduced in GSE158494 (Figures 1C,D), supporting the robustness and consistency of resistance-associated expression changes across experimental systems. We next summarized the patient-level progression characteristics within the TCGA-PRAD cohort. The unstratified distribution of progression-free interval (PFI) time and the proportion of progression events versus censored cases (Figure 1E) are shown for descriptive purposes to summarize cohort-level follow-up characteristics across 497 patients, while gene-specific stratified survival analyses are presented in subsequent sections. Major clinical features—including age at diagnosis, tumor status, and histological subtype—were further visualized using barplots and radar charts (Figures 1F–H), offering a detailed phenotypic profile of the cohort that will be leveraged for subsequent prognostic modeling. Finally, to assess the concordance of resistance-associated transcriptional changes across GEO datasets, we compared log2 fold-changes for overlapping DEGs. While substantial heterogeneity was observed at the individual gene level, the overall directionality of expression changes showed limited but detectable concordance between datasets (Figure 1I), reflecting both shared resistance-associated programs and context-dependent variability across experimental systems. Collectively, Figure 1 provides a comprehensive overview integrating GEO-derived resistance phenotypes with TCGA-PRAD clinical and molecular characteristics to establish the analytical framework for subsequent biomarker identification and functional prioritization.

### 3.2 Identification of shared docetaxel resistance–associated DEGs and functional annotation

**FIGURE 2:**
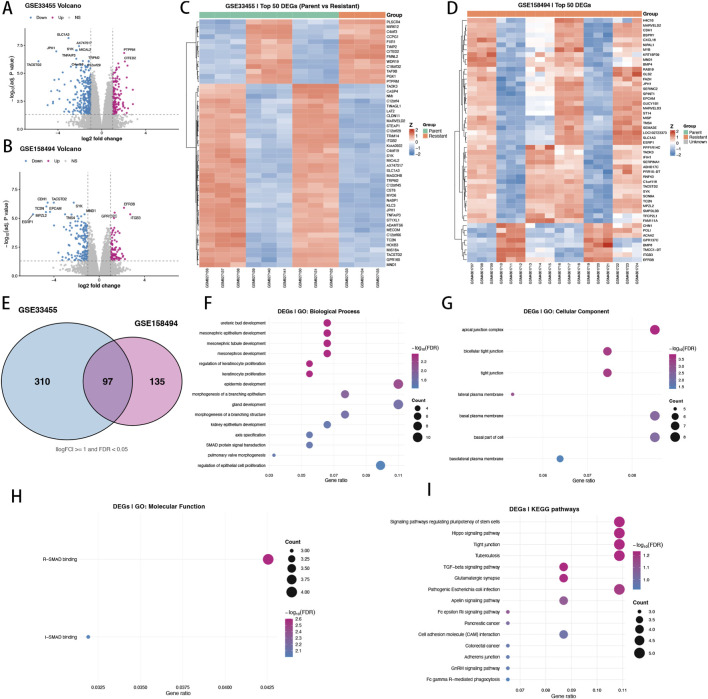
Identification of docetaxel resistance–related DEGs across two GEO cohorts and functional enrichment. **(A,B)** Volcano plots of DEGs in GSE33455 and GSE158494 (Resistant vs. Parent). Red, upregulated; blue, downregulated; gray, not significant (|log2FC| ≥ 1, FDR <0.05). **(C,D)** Heatmaps of the top 50 DEGs in GSE33455 and GSE158494. Expression values were Z-score scaled by gene; samples are annotated as Parent or Resistant. **(E)** Venn diagram showing the overlap of DEGs between the two datasets; 97 shared genes were retained as the consensus resistance signature. **(F–H)** GO enrichment of the 97 shared DEGs (BP, CC, and MF). **(I)** KEGG pathway enrichment of the 97 shared DEGs.

We next performed differential expression analyses in each GEO cohort to define transcriptional alterations associated with docetaxel resistance. Volcano plots revealed substantial gene expression remodeling in both datasets (Figures 2A,B). Using a unified threshold (|log2FC| ≥ 1 and FDR <0.05), we identified 407 DEGs in GSE33455 and 232 DEGs in GSE158494, indicating both common and cohort-specific resistance-linked programs. Heatmaps of the top 50 DEGs further demonstrated coherent expression patterns that distinguish parental from resistant states, supporting the robustness of the resistance phenotype at the transcriptomic level (Figures 2C,D). To derive a conservative and reproducible resistance-associated gene set, we intersected DEGs across the two cohorts and obtained 97 shared genes (Figure 2E). This consensus gene set was subsequently used as the primary candidate feature pool for downstream prognostic modeling in TCGA-PRAD. Functional enrichment analysis of the 97 shared DEGs highlighted biological themes related to epithelial organization and signaling regulation. GO enrichment indicated overrepresentation of processes linked to tissue/epithelial development and morphogenesis (BP), as well as cellular components associated with apical/basolateral plasma membrane and junctional complexes (CC) (Figures 2F,G). At the molecular function level, SMAD binding terms were enriched (MF), suggesting potential involvement of TGF-β/SMAD-associated transcriptional regulation in resistance-associated reprogramming (Figure 2H). Consistently, pathway analysis further implicated junction-related and signaling pathways, including tight junction and Hippo/TGF-β-related programs (Figure 2I). Collectively, these results support the presence of a reproducible docetaxel resistance transcriptional signature across independent GEO cohorts, providing a biologically interpretable and modeling-ready gene set for subsequent clinical outcome prediction.

### 3.3 Prognostic screening and derivation of a compact 3-gene signature in TCGA–PRAD

**FIGURE 3:**
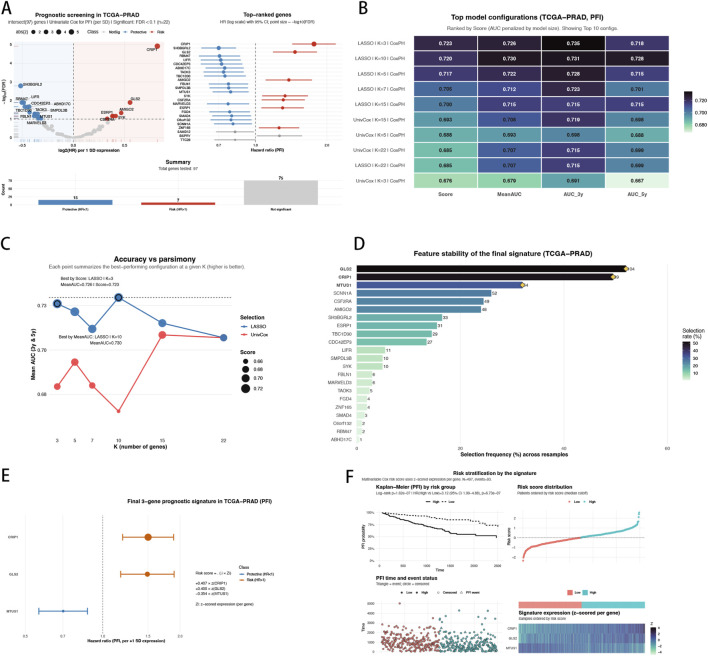
Prognostic screening and derivation of a compact 3-gene signature in TCGA–PRAD (PFI). **(A)** Univariate Cox regression across the 97 consensus docetaxel-resistance genes in TCGA–PRAD using per-SD (z-scored) expression, highlighting significant prognostic candidates by BH-FDR and their direction of effect (risk vs. protective). **(B)** Heat-table summarizing the top 10 model configurations ranked by a parsimony-aware Score (penalizing larger signatures), reporting Score, MeanAUC, and time-point AUCs. **(C)** Accuracy–parsimony landscape showing how discrimination changes with gene-set size (K) and feature-selection strategy (UnivCox ranking vs. LASSO ranking). **(D)** Feature stability under repeated resampling for the best-by-Score setting (LASSO + K = 3), reporting selection frequency across resamples and highlighting the most consistently selected genes (CRIP1, GLS2, and MTUS1). **(E)** Multivariable Cox model for the final 3-gene signature, visualizing hazard ratios with 95% CI and presenting the risk-score formula based on z-scored gene expression. **(F)** Risk stratification based on the 3-gene risk score, including risk-score distribution, event/censoring pattern, and Kaplan–Meier curves comparing high-vs. low-risk groups.

To translate the GEO-derived docetaxel-resistance program into clinically relevant prognostic markers, we evaluated 97 consensus resistance-related genes in TCGA–PRAD (n = 497) using univariate Cox regression with progression-free interval (PFI) as the endpoint. Using per-SD (z-scored) expression to ensure comparability across genes, we identified 22 prognostic candidates passing BH-FDR <0.10 (Figure 3A), indicating that a substantial subset of the resistance-associated transcriptome is also linked to clinical progression risk. We then implemented a systematic model-selection procedure to balance predictive accuracy with model sparsity. Candidate features were ranked using either univariate Cox significance or LASSO-based importance, and top-K gene sets were used to fit Cox proportional hazards models. Across varying K values, discrimination improved only modestly with larger signatures, whereas compact solutions achieved near-optimal performance (Figure 3C). When configurations were ranked by a parsimony-aware Score, LASSO + K = 3 emerged as the best overall setting, while LASSO + K = 10 provided the best MeanAUC solution (Figures 3B,C). To further justify model compactness, we assessed feature stability under repeated resampling for the best-by-Score configuration. This analysis demonstrated that CRIP1, GLS2, and MTUS1 were the most consistently selected features when restricting the model to K = 3 (Figure 3D), supporting their robustness as a stable prognostic core. Finally, a multivariable Cox model incorporating these three genes was fitted to derive a risk score as a weighted linear combination of z-scored expression values (Figure 3E). The resulting 3-gene risk score effectively stratified patients into distinct prognostic subgroups, showing significant separation in PFI outcomes between high- and low-risk strata (Figure 3F).

### 3.4 Cross-cohort validation and pathway context of the CRIP1–GLS2–MTUS1 signature

**FIGURE 4:**
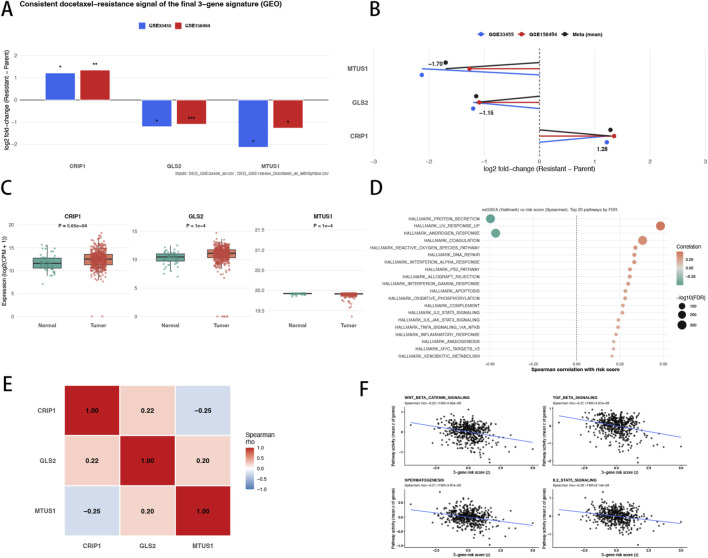
Cross-cohort validation and functional annotation of the CRIP1–GLS2–MTUS1 signature. **(A)** Differential expression of CRIP1, GLS2, and MTUS1 between docetaxel-resistant and parental prostate cancer models in two independent GEO cohorts (GSE33455 and GSE158494), shown as log2 fold-change (Resistant − Parent) estimated by limma. **(B)** Forest plot summarizing cohort-specific effect sizes (log2 fold-change with 95% CI) and the pooled meta-effect across the two GEO cohorts. **(C)** Expression levels of CRIP1, GLS2, and MTUS1 in TCGA–PRAD primary tumors and normal prostate tissues based on STAR-count data after CPM normalization and log2 transformation. **(D)** Bubble plot showing Spearman correlations between the 3-gene signature risk score and Hallmark pathway activity scores in TCGA–PRAD; dot color indicates correlation direction/strength and dot size reflects multiple-testing adjusted significance. **(E)** Spearman correlation heatmap illustrating co-expression relationships among CRIP1, GLS2, and MTUS1 across TCGA–PRAD tumor samples. **(F)** Representative scatter plots of selected Hallmark pathways versus the signature risk score, with fitted trend lines and annotated correlation statistics.

To further validate the robustness of the final 3-gene signature and clarify its biological context, we performed cross-cohort consistency checks in docetaxel-resistance models and complementary analyses in TCGA–PRAD. In the two independent GEO cohorts (GSE33455 and GSE158494), CRIP1, GLS2, and MTUS1 exhibited concordant expression shifts between resistant and parental conditions (Figure 4A), indicating that the final prognostic core is not merely a product of TCGA-based modeling but is also embedded in the transcriptional program accompanying acquired docetaxel resistance. A pooled summary of cohort-level effects further supported directionally consistent regulation across datasets (Figure 4B), reinforcing that these three genes capture a reproducible resistance-linked signal rather than cohort-specific noise. We next assessed whether the three genes retained coherent expression patterns in clinical specimens. Using TCGA–PRAD STAR-count data, all three genes showed clear tumor–normal expression differences (Figure 4C), supporting their relevance in the clinical setting. To connect this compact signature to broader transcriptional states, we related the continuous 3-gene risk score to Hallmark pathway activity across TCGA–PRAD samples. Multiple pathways displayed significant correlations with risk (Figure 4D), suggesting that the signature aggregates information from coordinated biological programs instead of isolated single-gene effects. Finally, co-expression analysis revealed structured relationships among CRIP1, GLS2, and MTUS1 (Figure 4E), and representative pathway–risk scatter plots illustrated the continuous coupling between risk and pathway activation at the individual-sample level (Figure 4F). Collectively, Figure 4 provides convergent evidence that the final CRIP1–GLS2–MTUS1 signature is reproducibly linked to docetaxel resistance across experimental cohorts and reflects coherent pathway-level remodeling in TCGA–PRAD.

### 3.5 Experimental validation of CRIP1 in docetaxel-resistant prostate cancer cells

**FIGURE 5:**
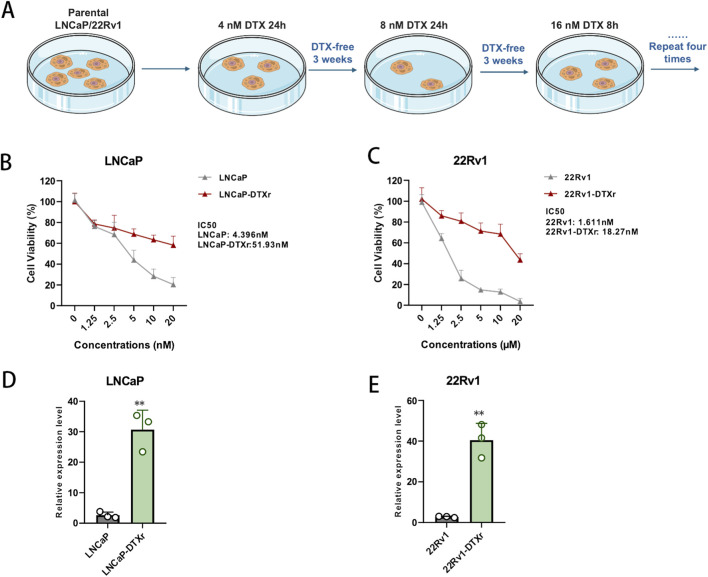
Establishment of docetaxel-resistant prostate cancer cells and CRIP1 upregulation. **(A)** Schematic workflow for generating docetaxel-resistant LNCaP and 22Rv1 sublines by stepwise docetaxel (DTX) exposure and recovery. **(B,C)** CCK-8 dose–response curves showing docetaxel sensitivity in parental LNCaP/22Rv1 cells and their resistant sublines (LNCaP-DTXr, 22Rv1-DTXr); IC50 values increase from 4.396 nM to 51.93 nM (LNCaP vs. LNCaP-DTXr) and from 1.611 nM to 18.27 nM (22Rv1 vs. 22Rv1-DTXr). **(D,E)** qRT–PCR analysis of CRIP1 mRNA expression in parental and docetaxel-resistant cells, demonstrating significantly higher CRIP1 levels in LNCaP-DTXr and 22Rv1-DTXr compared with their parental counterparts. Data are presented as mean ± SD from three independent experiments; P < 0.01 by unpaired two-tailed t-test.

Given that CRIP1 emerged as a high-priority candidate from the integrated bioinformatics pipeline—showing robust association with docetaxel resistance in GEO cohorts, prognostic relevance in TCGA–PRAD, and inclusion in the final three-gene signature—we selected CRIP1 for experimental validation in prostate cancer cell models. To mimic clinical acquisition of docetaxel resistance, we established docetaxel-resistant LNCaP and 22Rv1 sublines by exposing parental cells to stepwise increasing concentrations of docetaxel followed by recovery periods in drug-free medium. CCK-8 assays confirmed a clear shift in drug sensitivity: compared with their respective parental cells, LNCaP-DTXr and 22Rv1-DTXr cells maintained significantly higher viability across a range of docetaxel doses, with IC50 values increasing from 4.396 nM to 51.93 nM in LNCaP and from 1.611 nM to 18.27 nM in 22Rv1, respectively. These data demonstrate that the selected treatment schedule successfully generated stable docetaxel-resistant prostate cancer cell models (Figures 5A–C). We next examined whether CRIP1 expression was altered in the resistant phenotype. Quantification of CRIP1 mRNA levels revealed markedly higher expression in LNCaP-DTXr and 22Rv1-DTXr cells compared with their parental counterparts, with consistent upregulation observed in both cell line pairs (Figures 5D,E). Together with the transcriptomic and prognostic evidence, these findings indicate that CRIP1 is not only enriched in docetaxel-resistant clinical tumors but is also strongly associated with the acquisition of docetaxel resistance *in vitro*, supporting its role as a key mediator linking malignant progression and chemoresistance in prostate cancer.

### 3.6 CRIP1 knockdown resensitizes docetaxel-resistant prostate cancer cells

**FIGURE 6:**
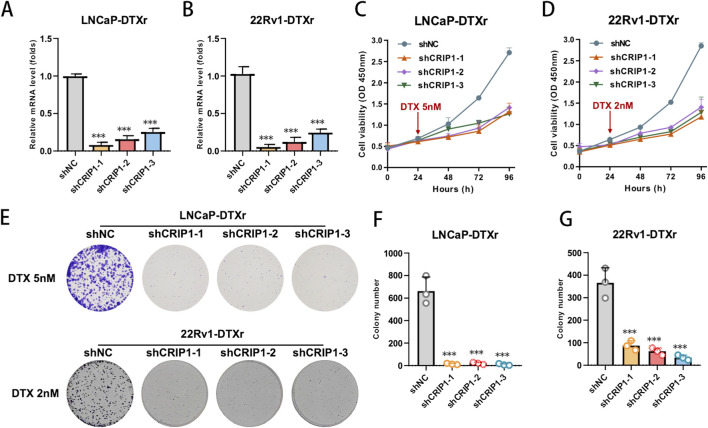
CRIP1 knockdown reverses docetaxel resistance and reduces clonogenic survival in resistant prostate cancer cells. **(A,B)** qPCR validation of CRIP1 knockdown in LNCaP-DTXr **(A)** and 22Rv1-DTXr **(B)** cells. Cells were transduced with a non-targeting control (shNC) or three independent shRNAs against CRIP1 (shCRIP1-1, shCRIP1-2, shCRIP1-3). CRIP1 mRNA levels were normalized to GAPDH and expressed relative to shNC, confirming efficient knockdown in both resistant sublines. **(C,D)** CCK-8 assay showing that CRIP1 knockdown restores docetaxel sensitivity. LNCaP-DTXr **(C)** and 22Rv1-DTXr **(D)** cells expressing shNC or shCRIP1 constructs were treated with docetaxel (5 nM for LNCaP-DTXr, 2 nM for 22Rv1-DTXr), and cell viability was monitored over 96 h. CRIP1-depleted cells exhibited markedly reduced viability compared with shNC after docetaxel exposure. **(E–G)** Clonogenic survival of resistant cells under docetaxel treatment after CRIP1 knockdown. **(E)** Representative colony formation images for LNCaP-DTXr and 22Rv1-DTXr cells transduced with shNC or shCRIP1 constructs and treated with docetaxel (5 nM or 2 nM, respectively). **(F–G)** Quantification of colony numbers in LNCaP-DTXr **(F)** and 22Rv1-DTXr **(G)** cells, showing significantly decreased clonogenic survival in all CRIP1 knockdown groups compared with shNC. Data are presented as mean ± SD from three independent experiments; statistical significance was assessed using an unpaired two-tailed t-test (***P < 0.001).

To test whether CRIP1 functionally contributes to the docetaxel-resistant phenotype, we performed loss-of-function experiments in LNCaP-DTXr and 22Rv1-DTXr cells. Three independent short hairpins targeting CRIP1 (shCRIP1-1, shCRIP1-2, shCRIP1-3) efficiently reduced CRIP1 mRNA expression in both resistant sublines compared with the non-targeting control (shNC), confirming robust knockdown at the transcript level (Figures 6A,B). We then evaluated the impact of CRIP1 depletion on docetaxel sensitivity and long-term survival. CCK-8 assays showed that CRIP1 knockdown markedly reduced cell viability following docetaxel treatment (5 nM for LNCaP-DTXr and 2 nM for 22Rv1-DTXr), indicating a clear resensitization of resistant cells to docetaxel (Figures 6C,D). Consistently, colony formation assays revealed that CRIP1-depleted cells formed substantially fewer colonies under docetaxel exposure than shNC controls in both resistant models (Figures 6E–G). Together, these results demonstrate that CRIP1 knockdown can partially reverse the docetaxel-resistant phenotype and attenuate the clonogenic capacity of prostate cancer cells, supporting a functional role for CRIP1 in maintaining chemoresistant growth.

### 3.7 CRIP1 knockdown attenuates migration of docetaxel-resistant prostate cancer cells

**FIGURE 7:**
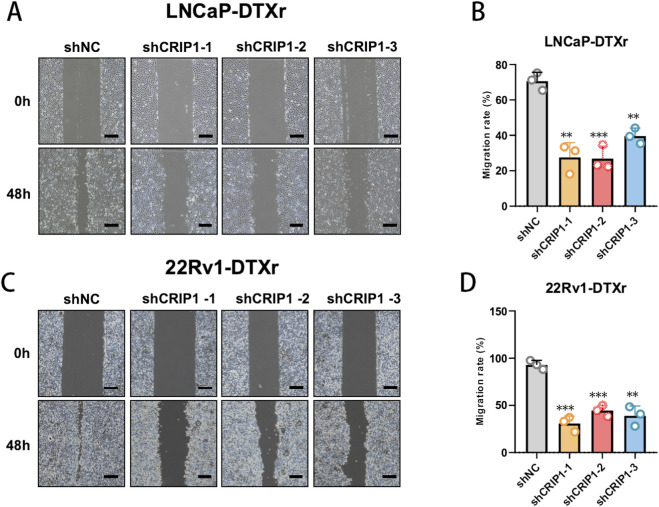
CRIP1 knockdown reduces migration of docetaxel-resistant prostate cancer cells. **(A,B)** Wound-healing assay in LNCaP-DTXr cells transduced with shNC or CRIP1 shRNAs, showing slower wound closure and reduced migration after CRIP1 depletion. **(C,D)** Wound-healing assay in 22Rv1-DTXr cells, with CRIP1 knockdown similarly impairing wound closure compared with shNC. Scale bars: 100 μm. Data are mean ± SD (n = 3); **P < 0.01, *P < 0.001 vs. shNC (unpaired two-tailed t-test).

To further characterize the impact of CRIP1 on malignant behavior, we examined cell migration using wound-healing assays in docetaxel-resistant models. In LNCaP-DTXr cells, CRIP1 knockdown markedly slowed wound closure compared with the shNC control, with all three shCRIP1 constructs consistently reducing the migrated area over 48 h (Figures 7A,B). A similar pattern was observed in 22Rv1-DTXr cells, where CRIP1-silenced groups showed substantially impaired wound closure relative to shNC (Figures 7C,D). These results indicate that CRIP1 is functionally required for the maintenance of docetaxel-resistant phenotypes in prostate cancer cells.

### 3.8 CRIP1 knockdown restores docetaxel-induced apoptosis in resistant prostate cancer cells

**FIGURE 8:**
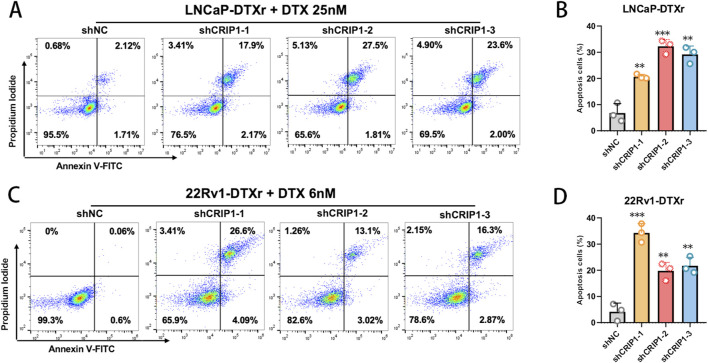
CRIP1 knockdown restores docetaxel-induced apoptosis in resistant prostate cancer cells. **(A)** Representative Annexin V–FITC/propidium iodide (PI) flow cytometry plots of LNCaP-DTXr cells transduced with shNC or CRIP1-targeting shRNAs (shCRIP1-1, shCRIP1-2, shCRIP1-3) and subsequently treated with 25 nM docetaxel for 24 h. CRIP1-silenced cells exhibited markedly increased early and late apoptotic populations compared with shNC. **(B)** Quantification of apoptotic rates in LNCaP-DTXr cells. All three CRIP1 shRNAs significantly enhanced docetaxel-induced apoptosis, indicating restored drug sensitivity. Data are shown as mean ± SD from three independent experiments. **(C)** Representative Annexin V–FITC/PI plots of 22Rv1-DTXr cells under docetaxel treatment (6 nM, 24 h) following CRIP1 knockdown. Similar to LNCaP-DTXr, depletion of CRIP1 robustly increased apoptosis compared with shNC. **(D)** Quantification of apoptotic rates in 22Rv1-DTXr cells. CRIP1 knockdown significantly promoted docetaxel-induced apoptosis across all shRNA constructs. Statistical analysis was performed using an unpaired two-tailed t-test (**P < 0.01, ***P < 0.001).

To determine whether the resensitization to docetaxel observed after CRIP1 knockdown was accompanied by enhanced cell death, we next assessed apoptosis in LNCaP-DTXr and 22Rv1-DTXr cells using Annexin V–FITC/PI staining. In LNCaP-DTXr cells, CRIP1 silencing with three independent shRNAs led to a marked increase in both early and late apoptotic fractions following exposure to docetaxel (25 nM, 24 h) compared with the shNC group (Figures 8A,B). A similar pattern was observed in 22Rv1-DTXr cells treated with docetaxel at 6 nM, where CRIP1-depleted cells consistently exhibited higher apoptotic rates than control cells (Figures 8C,D). These findings indicate that CRIP1 knockdown not only reduces the proliferative and clonogenic capacity of docetaxel-resistant prostate cancer cells but also restores docetaxel-triggered apoptosis, further supporting a survival-promoting role of CRIP1 in the chemoresistant state.

### 3.9 CRIP1 knockdown enhances docetaxel-induced ICD

**FIGURE 9:**
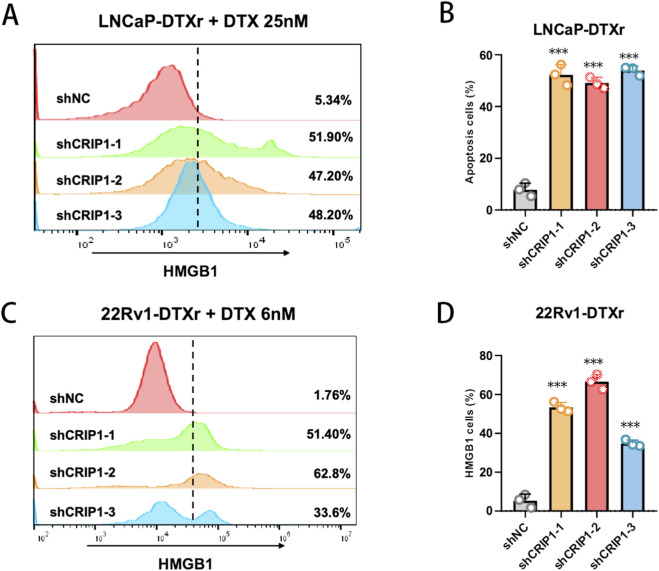
CRIP1 knockdown enhances docetaxel-induced immunogenic cell death in resistant prostate cancer cells. **(A,B)** Flow cytometry of HMGB1 in LNCaP-DTXr cells transduced with shNC or CRIP1 shRNAs and treated with docetaxel (25 nM, 24 h), showing increased HMGB1-positive cells after CRIP1 depletion. **(C,D)** HMGB1 analysis in 22Rv1-DTXr cells treated with docetaxel (6 nM, 24 h), with CRIP1 knockdown similarly elevating HMGB1-positive fractions compared with shNC. Data are mean ± SD (n = 3).

Given the observed increase in docetaxel-induced apoptosis after CRIP1 knockdown, we next examined whether CRIP1 also affects ICD (ICD). Using HMGB1 as a surrogate ICD marker, flow cytometry demonstrated that CRIP1 depletion in LNCaP-DTXr cells markedly increased HMGB1-positive populations following docetaxel treatment (25 nM, 24 h) compared with shNC control (Figures 9A,B). A similar enhancement of HMGB1 release was observed in 22Rv1-DTXr cells exposed to docetaxel at 6 nM, with all three shCRIP1 constructs significantly elevating the fraction of HMGB1-positive cells and shCRIP1-2 showing the strongest effect (Figures 9C,D). These findings suggest that CRIP1 knockdown not only restores docetaxel-induced apoptosis but also amplifies ICD-associated signaling, potentially rendering docetaxel-resistant prostate cancer cells more immunogenic under chemotherapy stress.

### 3.10 CRIP1 knockdown restores docetaxel sensitivity in LNCaP-DTXr xenograft models

**FIGURE 10:**
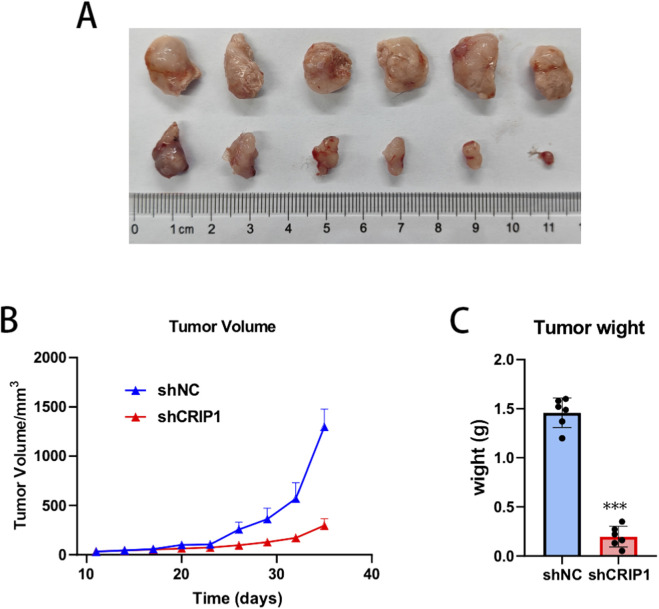
CRIP1 knockdown restores docetaxel sensitivity in LNCaP-DTXr xenograft tumors. **(A)** Representative images of xenograft tumors derived from LNCaP-DTXr cells stably expressing shNC or shCRIP1 at the experimental endpoint. All mice were treated with docetaxel (10 mg/kg) according to the same dosing schedule. **(B)** Tumor growth curves of LNCaP-DTXr xenografts during docetaxel treatment. CRIP1 knockdown significantly suppressed tumor growth compared with shNC controls, indicating enhanced chemosensitivity. Tumor volume was measured every 3 days. Data are presented as mean ± SD. **(C)** Final tumor weights at sacrifice. Tumors from the shCRIP1 group were significantly lighter than those from the shNC group, consistent with restored docetaxel responsiveness *in vivo*. Statistical analysis was performed using an unpaired two-tailed t-test. **P < 0.001.

To validate the functional role of CRIP1 in docetaxel resistance *in vivo*, LNCaP-DTXr cells stably expressing shNC or shCRIP1 were implanted to establish xenograft tumors, and mice in both groups were treated with docetaxel at 10 mg/kg. At the experimental endpoint, xenografts derived from shCRIP1-expressing cells were visibly smaller than those from the shNC group (Figure 10A). Consistently, longitudinal tumor growth analysis revealed that CRIP1 knockdown significantly suppressed tumor expansion under docetaxel treatment, whereas shNC tumors continued to grow despite chemotherapy (Figure 10B). Quantitative assessment further demonstrated that final tumor weights were markedly reduced in the shCRIP1 group compared with controls (Figure 10C). Collectively, these results demonstrate that CRIP1 depletion restores docetaxel sensitivity in docetaxel-resistant prostate cancer xenografts, providing *in vivo* evidence that CRIP1 contributes to the maintenance of chemoresistant phenotypes.

## 4 Discussion

In this study, we combined multi-cohort transcriptomic profiling, systematic prognostic modeling, and functional experiments to identify and validate a compact CRIP1–GLS2–MTUS1 signature associated with docetaxel resistance and progression-free interval in prostate cancer. By intersecting DEGs from two independent docetaxel-resistant GEO cohorts and then projecting this resistance program into TCGA-PRAD, we showed that a subset of these resistance-linked genes also carries strong prognostic information for PFI. Through a parsimony-aware grid search, we derived a three-gene Cox model that retained robust prognostic discrimination while minimizing model complexity, and we further demonstrated that CRIP1 is not only enriched in resistant cells but functionally promotes chemoresistance, proliferation, migration, and apoptosis evasion in docetaxel-resistant prostate cancer cells.

Compared with previously reported multi-gene signatures for prostate cancer prognosis, which often derive from bulk tumor cohorts without explicit linkage to therapy-induced resistance, our modeling strategy starts from experimentally defined docetaxel-resistant systems and then validates in clinically annotated patients. Many established genomic classifiers (e.g., Decipher-like panels) emphasize metastatic risk and biochemical recurrence, but do not explicitly integrate drug-resistance biology or chemotherapeutic exposure in their design. By contrast, our pipeline uses overlapping resistance-associated DEGs as the initial feature pool, screens them for PFI relevance, and then constrains the final model to a very small set of genes that are reproducibly dysregulated in docetaxel-resistant preclinical models and clinically prognostic in TCGA–PRAD. This design provides a mechanistic bridge between taxane resistance and clinical progression, and may be particularly relevant for castration-resistant or treatment-escalation settings where taxane exposure is common.

Within the three-gene core, CRIP1 emerged as a central effector that connects docetaxel resistance with more aggressive cellular phenotypes. Previous work has implicated CRIP1 as an oncogenic factor in several solid tumors (Liu et al., 2023; Wang et al., 2022; Wu et al., 2023). In breast cancer, the prognostic direction of CRIP1 appears context-dependent, with reports linking CRIP1 expression to clinicopathologic features and patient outcome in a subtype- and cohort-specific manner (Ludyga et al., 2013). In colorectal cancer, CRIP1 overexpression suppresses apoptosis and supports resistance to 5-fluorouracil, providing direct evidence that this protein can mediate chemotherapy tolerance (Zhang et al., 2019). Independent functional studies have shown that CRIP1 silencing reduces migration and invasion capacity in colorectal cancer models, consistent with a broader role in sustaining pro-metastatic behavior (He et al., 2017; He et al., 2019). Our data extend these observations to prostate cancer by demonstrating that CRIP1 is upregulated in docetaxel-resistant LNCaP-DTXr and 22Rv1-DTXr cells (O'Neill et al., 2011), that its knockdown restores docetaxel sensitivity in viability and clonogenic assays, and that it attenuates the enhanced migratory capacity acquired during resistance. Taken together, these lines of evidence support CRIP1 as a context-agnostic driver of chemoresistant and pro-malignant phenotypes across multiple tumor types (Ye et al., 2025). Because our functional evidence is based on loss-of-function approaches, the present study supports a role for CRIP1 in maintaining established docetaxel resistance rather than demonstrating that CRIP1 is sufficient to drive resistance acquisition.

Within the prognostic signature, GLS2 and MTUS1 exhibit complementary and predominantly protective roles. In contrast, elevated CRIP1 expression is associated with increased risk. Accordingly, in the composite risk model, CRIP1 contributes positively to progression-free interval (PFI) risk, whereas GLS2 and MTUS1 exert inverse (protective) effects, together shaping the overall risk score. This pattern is consistent with several reports describing GLS2 and MTUS1 as potential tumor-suppressive or differentiation-linked genes in other solid tumors, although their functions in prostate cancer remain comparatively underexplored (Hu et al., 2010; Suzuki et al., 2010; Ding et al., 2012; Zhao et al., 2015). From a biological standpoint, GLS2, a mitochondrial glutaminase, has been implicated in regulating cellular metabolism and oxidative stress, while MTUS1 is known to encode ATIP proteins involved in microtubule dynamics and growth factor signaling (Hu et al., 2010; Suzuki et al., 2010; Rodrigues-Ferreira et al., 2009). It is plausible that, within the context of taxane-treated prostate cancer, loss or downregulation of such “brakes” may cooperate with high CRIP1 to promote survival under cytotoxic stress; however, this hypothesis will require direct mechanistic dissection in future work.

A notable aspect of our study is the link between CRIP1 and immunogenic cell death (ICD)–related signaling. ICD is characterized by the emission of damage-associated molecular patterns (DAMPs), including HMGB1 release, ATP secretion, and calreticulin exposure, which can convert dying tumor cells into an *in situ* vaccine and prime adaptive immune responses (Apetoh et al., 2007; Obeid et al., 2007; Garg et al., 2012; Wang et al., 2018). Chemotherapeutic agents such as anthracyclines and certain taxanes can induce ICD under specific conditions, thereby providing both direct cytotoxic and immunostimulatory benefits (Apetoh et al., 2007; Hodge et al., 2013). Our flow-cytometric analyses show that CRIP1 knockdown not only increases docetaxel-induced apoptosis but also augments HMGB1-positive cells in docetaxel-resistant LNCaP-DTXr and 22Rv1-DTXr models, suggesting that CRIP1 downregulation may partially restore the immunogenic component of docetaxel-triggered cell death. Although our experiments were performed *in vitro* without immune cells, these findings raise the intriguing possibility that targeting CRIP1 could synergize with taxane chemotherapy by both resensitizing tumor cells and enhancing their capacity to engage antitumor immunity.

From a translational perspective, our work supports two complementary applications aligned with the theme of biomarker- and target-driven precision therapy. First, the CRIP1–GLS2–MTUS1 risk score provides a compact, interpretable signature that could help stratify patients by PFI risk and potentially identify those more likely to harbor a taxane-resistant transcriptional program. Because the model was trained on PFI and derived from resistance-linked genes, it may be particularly useful for flagging patients at risk of early progression under intensified systemic therapies. Second, CRIP1 itself emerges as an attractive therapeutic node. While no CRIP1-targeted agents are currently available, our data suggest that approaches such as RNA-based silencing, targeted protein degradation, or indirect pathway inhibition might be explored to weaken chemoresistant clones and to enhance docetaxel efficacy (Yan Y. et al., 2025). In a broader immuno-oncology context, combining CRIP1 inhibition with ICD-inducing chemotherapy and immune checkpoint blockade could, in principle, leverage restored ICD signaling to improve immunotherapy responsiveness in advanced prostate cancer, although this remains speculative and requires rigorous *in vivo* evaluation (Ghiringhelli and Rébé, 2024).

Several limitations should be acknowledged. First, although we integrated two docetaxel-resistant GEO datasets and a large TCGA–PRAD cohort, our analyses are still retrospective and constrained by the available data structure. The TCGA–PRAD cohort mainly represents primary, largely hormone-sensitive prostate tumors; docetaxel exposure was not part of the standardized treatment for these patients, and thus the PFI endpoint may not fully capture taxane-specific resistance dynamics. Second, our experimental validation was performed in 2 cell line pairs (LNCaP/22Rv1 and their docetaxel-resistant derivatives), which, while widely used, cannot recapitulate the full heterogeneity of clinical CRPC, including stromal interactions, androgen-receptor splice variants, and immune components. Third, we did not perform *in vivo* xenograft or orthotopic models to assess the impact of CRIP1 modulation on tumor growth, metastasis, or treatment response in an intact microenvironment. Finally, although we observed clear changes in HMGB1-defined ICD markers following CRIP1 knockdown and docetaxel treatment, we did not directly link these changes to functional immune readouts such as dendritic cell activation or T-cell priming.

Future work should therefore pursue several directions. On the clinical side, the CRIP1–GLS2–MTUS1 signature warrants validation in independent, treatment-annotated cohorts of prostate cancer, particularly in patients receiving taxane-based chemotherapy, to further establish its predictive and prognostic relevance across disease stages. It should also be noted that the resistance-associated signature in this study was derived from AR-null docetaxel-resistant models, whereas functional validation was primarily conducted in AR-positive systems. Given the biological differences between AR-driven and AR-independent prostate cancer, resistance mechanisms may not be fully conserved across these contexts, and the generalizability of CRIP1-associated resistance programs to AR-null disease states requires further investigation. In addition, the functional experiments in this study were specifically designed to interrogate CRIP1-dependent modulation of cellular responses to docetaxel exposure rather than the intrinsic cytotoxic effects of CRIP1 perturbation alone, which should be taken into account when interpreting the observed sensitization phenotypes. Finally, CRIP1 expression was primarily evaluated at the transcriptomic level in this study; while protein-level validation would further refine mechanistic interpretation, the integrative transcriptomic and functional analyses presented here demonstrate that modulation of CRIP1 expression is sufficient to influence docetaxel response phenotypes, supporting its functional relevance in resistance maintenance. From a mechanistic perspective, integrative multi-omics approaches—including phosphoproteomics, metabolomics, and single-cell or spatial transcriptomics—could further delineate the regulatory networks upstream and downstream of CRIP1 and clarify how it interfaces with established taxane resistance pathways such as drug efflux, microtubule remodeling, DNA damage responses, and AR signaling. Importantly, given the current lack of direct CRIP1 inhibitors, future work could explore more immediate therapeutic strategies, such as developing targeted protein degraders (e.g., PROTACs) against CRIP1 or identifying and inhibiting critical downstream effector pathways mediating CRIP1-associated docetaxel resistance.

In summary, by harnessing both AI-assisted bioinformatics and traditional wet-lab experimentation, we identify a three-gene CRIP1–GLS2–MTUS1 signature that links taxane-resistant transcriptional states to PFI in prostate cancer and functionally establish CRIP1 as a driver of docetaxel resistance, malignant behavior, and dampened ICD. Our findings nominate CRIP1 as a promising biomarker and potential therapeutic target in taxane-treated prostate cancer and illustrate a scalable strategy for discovering clinically actionable biomarkers at the interface of resistance biology and ICD.

## 5 Conclusion

In conclusion, by integrating docetaxel-resistant CRPC transcriptomic profiles with TCGA–PRAD PFI data, we defined a concise three-gene signature (CRIP1–GLS2–MTUS1) that links taxane-resistance–associated programs to clinical progression risk. Functional experiments confirmed that CRIP1 is upregulated in docetaxel-resistant prostate cancer cells and that its knockdown restores docetaxel sensitivity while attenuating proliferation, migration, and promoting apoptosis and HMGB1-associated ICD. These findings support the 3-gene signature as a practical prognostic tool and nominate CRIP1 as a promising therapeutic vulnerability in taxane-treated or castration-resistant prostate cancer, meriting further validation in independent cohorts and *in vivo* models.

## Data Availability

The original contributions presented in the study are publicly available. This data can be found here: The transcriptomic datasets analyzed during the current study are available in the Gene Expression Omnibus (GEO) repository (https://www.ncbi.nlm.nih.gov/geo/) under accession numbers GSE33455 and GSE158494, and The Cancer Genome Atlas (TCGA) database (https://portal.gdc.cancer.gov/) under project ID TCGA-PRAD.
